# Impact of the anatomical location, alcoholism and smoking on the prevalence of advanced oral cancer in Brazil

**DOI:** 10.4317/medoral.22318

**Published:** 2018-04-24

**Authors:** Natanael-Victor-Furtunato Bezerra, Karla-Lorene-de França Leite, Mariana-Marinho-Davino de Medeiros, Mariana-Leonel Martins, Andrea-Medeiros-Rodrigues Cardoso, Pollianna-Muniz Alves, Wilton-Wilney-Nascimento Padilha, Yuri-Wanderley Cavalcanti

**Affiliations:** 1 DDS, PhD, Clinical and Social Dentistry Department. Health Sciences Center. Federal University of Paraiba – Campus I, Cidade Universitária. João Pessoa-PB

## Abstract

**Background:**

To evaluate the prevalence of oral cancer in Brazil according to the clinical stage, anatomical location, alcoholism and smoking.

**Material and Methods:**

Data referring to 31,217 cases of oral cancer, from 2000 to 2010, were obtained from the Integrator Module of the Hospital Registry of Cancer. Inconsistent data (“non-classified” cases) was eliminated and 21,160 cases were analyzed. The frequency distribution according to clinical stage, anatomical location, alcoholism and smoking was analyzed descriptively and through a binary logistic regression model (α<0.05). The clinical stage (dependent variable) was dichotomized in early stage (I and II) or advanced stage (III and IV). The year of diagnosis, anatomical location and deleterious habits (alcoholism and smoking) were considered independent variables.

**Results:**

The most frequent characteristics were: oropharynx location (n=3856, 18.41%), clinical stage IV (n=11924, 56.09%) and combined use of alcohol and tobacco (n=19226; 61.59%). The year 2009 (*p*<0.01, PR = 1.162, CI-95%=1.053-1.283) and location at the base of tongue (*p*<0.01, PR = 2.485, CI-95% = 2.182-2.807) presented a higher prevalence ratio for advanced stage oral cancer. The combined use of alcohol and tobacco showed a higher prevalence rate for the advanced clinical stage of cancer (*p*<0.01, PR =1.449, CI-95%=1.382-1.520) if compared to individuals without habits, or just alcoholics.

**Conclusions:**

Higher prevalence of advanced stage of oral cancer is related to the localization at the base of the tongue and to the concomitant use of alcohol and tobacco. Therefore, it can be suggested that all these characteristics lead to a worse prognosis of oral cancer.

** Key words:**Oral cancer, neoplasm staging, alcoholism, tobacco use disorder.

## Introduction

The TNM staging system for the classification of malignant tumors distributes clinical stages of neoplasms at four levels (I, II, III and IV). Based on that classification, stages I and II are considered the initial stage, without regional and distant metastasis; whilst stages III and IV correspond to advanced stage, with regional or distant metastasis. Therefore, the determination of cancer staging is a very important tool for an adequate choice of antineoplastic treatment, as well as prediction of the prognosis of malignant neoplasm (1,2). In this sense, the higher the clinical stage of oral cancer, the worse is the prognosis and shorter the survival of affected individuals (2-5). Based on that, identifying factors related to the greater aggressiveness of neoplasms can contribute to the design of prevention strategies, with a view to the early diagnosis.

Among the factors related to the aggressiveness of malignant neoplasms, the anatomical location may be an aspect of relevance, since more apparent lesions are easily diagnosed, whilst others located in regions of lesser access or visualization are difficult to detect by the patient, or by the health professional (1). In addition, more vascularized regions (i.e.: mouth floor, the lateral border and the base of the tongue) allow greater dissemination of the disease, with a greater probability of development of regional metastasis (6,7). In this sense, these lesions can be diagnosed at advanced clinical stage (III or IV).

Alcoholism and smoking are deleterious habits identified as major risk factors for oral cancer, since their combination increases the chances of developing malignant neoplasms (5,8,9). Therefore, alcoholism and smoking habits are considered a public health problem, increasing the morbidity and mortality of cancer cases (9). In addition to risk factors such as sun exposure and eating habits, current studies report that types 16 and 18 of human papillomavirus (HPV) infection are related to the pathogenesis of oral cancer, mainly in the oropharynx (15-17). However, the impact of such habits on the prevalence of advanced stage cancer of the mouth and its relation with localization of cancer lesions still needs to be clarified.

Considering the above, the possible association of the severity of oral malignancies with the anatomical location and deleterious habits of alcoholism and smoking needs to be studied under a broader epidemiological aspect. Therefore, the present study aimed to evaluate the distribution of oral cancer cases according to the clinical stage, location and habits of alcoholism and smoking in Brazil, from 2000 to 2010.

## Material and Methods

A cross-sectional study was carried out based on the analysis of an information system that gathers data from all cases of cancer in Brazil. Data were collected from September to December 2016, using the “Integrator Module of the Hospital Registry of Cancer”, linked to the “National Cancer Institute of Brazil” (HRC Integrator - https://irhc.inca.gov.br/RHCNet/visualizaTabNetExterno.action). This information system retrieves some data collected by the physician in the event of diagnosis. In the present study, we used the following variables: year of diagnosis, clinical stage, deleterious habits and anatomical location of the oral cancer lesion.

The HRC Integrator provided data referring to 31,217 cases of oral cancer, treated in 228 hospital units in Brazil. Data referring to 26 federative units of Brazil were considered, excluding the state of São Paulo. According to data from the platform, hospitals in this state do not collect data on alcoholism and smoking at the time of diagnosis.

Included cases were classified according to the categories of the International Classification of Diseases for Oncology (ICD-10) (10), as follows: Lips (C00), Tongue base (C01), Tongue (C02), Gum (C03), mouth floor (C04), Palate (C05), Other non-specific parts of the mouth (C06), Parotid gland (C07), Others larger salivary glands (C08), Tonsils (C09) and Oropharynx (C10).

Preliminary database screening was performed to eliminate inconsistencies and un-relevant data. Cases registered as “without information” (code 9), “unconsidered” (code 8) and “without clinical stage” (code 0) were excluded from dataset. We did preserve only cases that had consistent data regarding clinical stage, deleterious habits, year of diagnosis and anatomical location of the lesion. The final sample value considered for statistical analysis was equal to 21,160 cases.

The descriptive analysis was performed to present the frequency distribution of clinical stages, anatomical locations and deleterious habits, according to the year of diagnosis. Then, a binary logistic regression model was elaborated, in which clinical stages (dependent variable) were dichotomized within initial stage (I and II - score 0) and advanced stage (III and IV - score 1). The year of diagnosis, location and deleterious habits of alcoholism (yes or no) and smoking (yes or no), reported by the patients at the time of diagnosis, were considered independent variables.

Data were tabulated and analyzed statistically in the Statistical Package for Social Sciences software (SPSS, v. 20, IBM, Chicago, IL). The input method of Backward:Wald and the significance level of 10% were considered as selection form for insertion of independent variables for model assembly in primary bivariate analysis. The adjustment of the model was performed, considering the Hosmer-Lemeshow goodness adjustment (*p*>0.05), where the model was considered properly adjusted (*p*=0.146). The prevalence ratio and confidence interval values were defined for the interpretation of results, considering the level of significance of 5% (α<0.05).

## Results

The most frequent anatomical sites diagnosed with oral cancer were Oropharynx (n=5748, 18.41%), followed by tongue (n=5600, 17.94%) and other parts of the mouth (n=3917, 12.55%) ( Table 1). However, the tongue considered as a whole (Tongue + Tongue base), was more frequent among oral cancer sites (28.62%, n=8934). In addition, the greater line slope on Figure 1 indicates a higher trend of increase for cases of oral cancer located in the oropharynx and in the tongue.

**Table 1:**
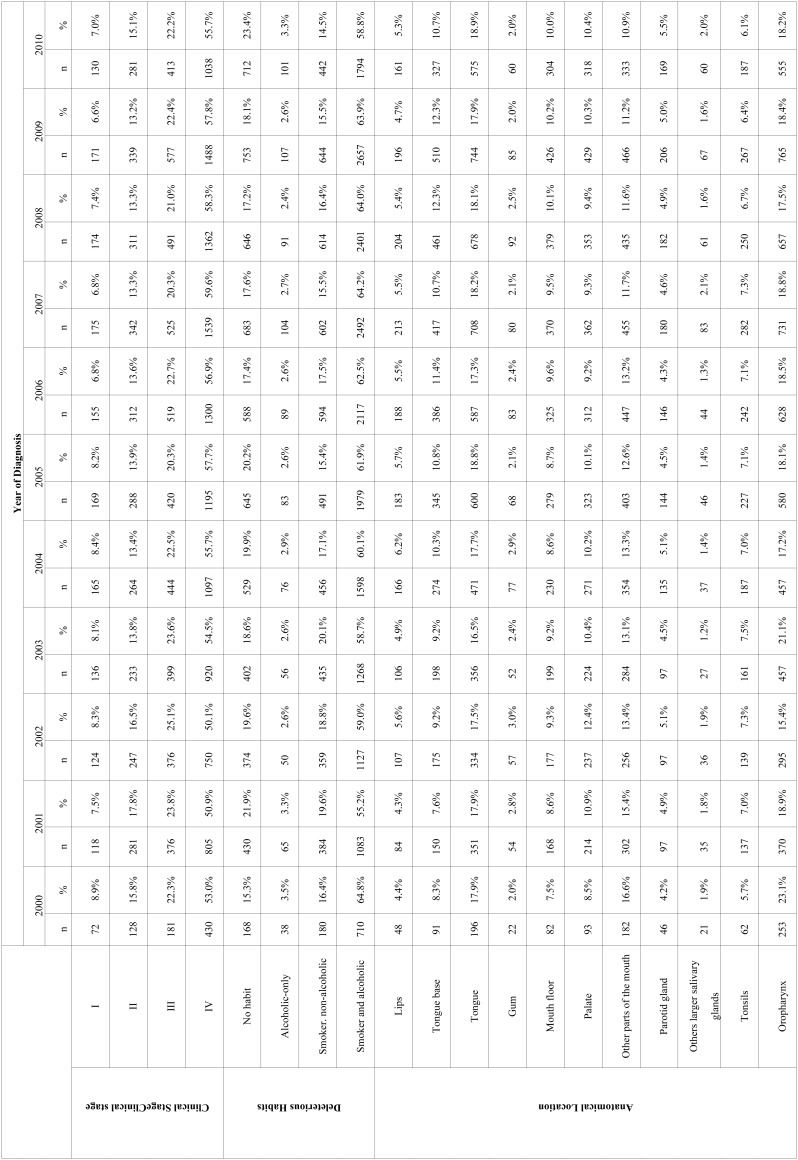
Frequency distribution of Clinical Stage, Deleterious Habits and Anatomical Location of oral cancer lesions according to the year of diagnosis.

Individuals diagnosed with oral cancer presented higher frequency of clinical stage IV (56.09%, n = 11924) ( Table 1). Among different clinical stages, there was a greater tendency of increase for cases of oral cancer in individuals affected by stage IV.

Individuals who self-declared alcoholics and smokers had higher prevalence of cancer (61.59%, n=19226) at the time of diagnosis. In addition, it was observed a greater tendency for the increase of oral cancer cases in alcoholics and smokers individuals.

Considering the whole period from 2000 to 2010, the prevalence of oral cancer in the advanced clinical stages (n=16545; 78.19%) was higher than that observed for the initial stages (n=4615; 21.81%). There was a higher prevalence rate for advanced clinical stage (III and IV) in 2001, 2007 and 2009, compared to the year 2000.

Compared to the cases diagnosed in lip, higher prevalence ratio for advanced clinical stage (III and IV) was verified for tongue base, gum, tonsils, oropharynx and other parts of the mouth (*p* <0.05 –  Table 2).

**Table 2:**
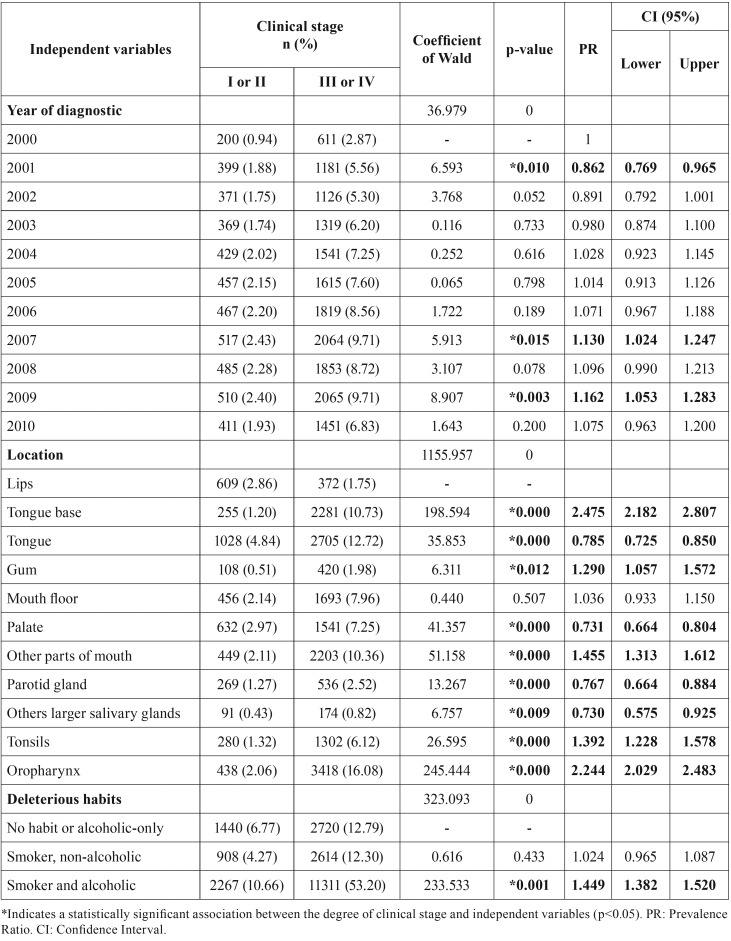
Distribution of components of the sample (n, %) according to the degree of clinical stage of the lesion (I/II or III/IV), year of diagnosis, anatomical location, and deleterious habits declared by the individuals at the time of diagnosis. Binary logistic regression model for association between clinical stage degree (I/II or III/IV) and independent variables analyzed (year of diagnosis, location and deleterious habits of the patient).

With regards to the analysis of deleterious habits ( Table 2), self-declared smoking-only individuals did not present a statistically significant prevalence ratio relative to non-alcoholics and non-smokers (*p*=0.433). However, a higher prevalence of oral cancer in the advanced clinical stage (grades III and IV) was observed among individuals who had concomitant use of alcohol and tobacco (*p*<0.01 –  Table 2).

## Discussion

According to the present study, the distribution of oral cancer in Brazil shows a high prevalence of advanced stage (grade IV), with neoplastic lesions in tongue (tongue + tongue base) and self-declared alcoholics and smokers. This profile confirms the previous findings in the literature (1,2,5,9,11,12) and shows up the greater aggressiveness of oral cancer, especially when located at the base of the tongue, in alcoholics and smokers (5,7,8,9,13).

A greater prevalence of oral cancers diagnosed in advanced clinical stage (III or IV) was observed in the year 2009, when compared to the year 2000. It is known that the year of registration does not explain the prevalence or evolution of cancer lesions. However, the analysis of the year using the binary logistic regression model shows a significant increase in the diagnosis of these lesions over the past few years. In Brazil, this increase in oral cancer registries may be related to the incentive of the National Oral Health Policy (Smiling Brazil) to the implantation and expansion of the network. In addition, there was a greater registry of cancer cases by the health information systems from the Ministry of Health, more specifically, the HRC Integrator (14).

The lip is the location of the mouth more accessible, visible and easy to identify any change in normality. Therefore, this location probably has a higher prevalence of early diagnosis (1). Due to these factors, the lip location was used as a comparison parameter in the logistic regression model used in this study. In this way, if compared to the lip, the base of the tongue presented a higher prevalence ratio for advanced cases. This can be justified by the lower visibility and access to this region for recognition of the lesion and, consequently, an early diagnosis. Most studies show a higher aggressiveness of the cancer located at the tongue base (6,12), due to their susceptibility to increased exposure to carcinogens (alcohol and tobacco), as well as the greater probability of involvement and the presence of cervical metastases due to its high vascularization (6,7).

Cancer located in the oropharynx presented the second highest prevalence ratio for advanced stage neoplasms compared to the lip. Similarly to the tongue base, the oropharynx corresponds to an area of difficult access and visibility (1). Recent studies point out to an increase in the incidence of oral cancer cases located in the oropharynx, usually associated with human papillomavirus (HPV) infection (1,2,9,11). Recent studies pointed out HPV has increased its importance on the pathogenesis of oral cancer, since anti-alcoholism and anti-tobacco awareness campaigns have successfully reduced the prevalence of such habits among youngers (15,16). Additionally, new cases of oral cancer associated with HPV has increased exponentially with a global prevalence ranging from 16 to 37% (16). Among young individuals, associated HPV infection present favorable prognosis due to the better overall health and lower mutation burden (17). Still, the literature is controversial with regards to the association of HPV infection and advanced clinical stage of oral cancer (9). Thus, more studies are needed to elucidate the association between HPV infection and increased aggressiveness of neoplasms located in the oropharynx.

Individuals diagnosed with oral cancer who did not present any deleterious habits and those who declared themselves only alcoholics were parameters of comparison in the present study. Alcoholics were put together to individuals with no deleterious habits because the literature reported that alcoholism alone is not associated with advanced stage of oral cancer (13,18). For individuals who have never been exposed to deleterious habits of alcoholism and smoking, the development of cancer can be attributed to other carcinogenic factors such as nutrition, viruses, immunosuppression and genetic predisposition. In the case of lip cancer, chronic sun exposure is considered the main risk factor (4,9).

The present study did not observe a statistically significant difference for the prevalence of advanced cases of oral cancer in self-declared smokers-only, compared to individuals without deleterious habits, or alcoholic-only. However, one study found greater aggressiveness of cancer in smokers (19). The combination of deleterious habits of alcoholism and smoking may increase the prevalence of advanced clinical stage, as observed in the present study. This finding ratifies the literature, showing greater aggressiveness of oral lesions in alcoholic and smoker individuals (5,7-9,13).

A higher aggressiveness of oral cancer in alcoholics and smokers may occur due to increased cell membrane permeability caused by ethanol and, consequently, exposure of intracellular content to tobacco as carcinogenic agent (20). Alcoholism and smoking induce decreased expression of Interleukin 18 (IL-18) (21) and DDX3 protein (22), which regulate the cell cycle and progression of malignant neoplasm. In addition, the COX-2 inflammatory marker, related to the aggressiveness of neoplasms, is more frequent in smokers, when compared to non-smokers (19).

The present study suggests an association between the higher prevalence of oral cancer in the advanced clinical stage with the anatomical site of the lesion, mainly in the tongue base, and deleterious habits of smoking and alcoholism. These two major risk factors are likely to increase the rate of injury progression (5,8,9,13). However, the methodology used in present study does not allow a cause-effect prediction, since analyses are based on a health information system (HRC Integrator). As discussed, data from this study are consistent with the literature, which records the increase on the prevalence of advanced-stage lesions, as well as elucidates the effect through biological mechanisms. However, the neglect of individuals with their own health and waiting time from detection of the first signal to confirm diagnosis may also contribute to a higher prevalence of advanced stage lesions (4,5,23).

The record of the increased prevalence of advanced-stage oral cancers is evident; however, these are not all cases existing in Brazil from 2000 to 2010. The limitation of the use of secondary data occurs because data may have been lost or not indexed to the platform. The inconsistency of data after 2010 can be verified in figures 1 to 3, in which the prevalence and insignificant data are observed after 2011. Based on that, data collection was carried out up to 2010 due to the inconsistency of data for the following years (2011 to 2017). Therefore, the HRC Integrator must be updated in order to correctly perform its function. These limitations; however, do not compromise the inferences observed in the study, being a mechanism for planning public policies directed to the population subject to risk factors related to oral cancer.

The lack of data from the state of São Paulo regarding alcohol and smoking habits in the HRC Integrator is a limitation of this study. Although the state of São Paulo computes the higher prevalence of cases of oral cancer, the influence of alcoholism and smoking cannot be evaluated in the present study. However, this lack of data regarding the state of São Paulo does not compromise the conclusions of this study, which confirm the location of the malignant neoplasm and the combination of alcoholism and smoking habits as the main risk factors for greater aggressiveness of oral cancer.

Higher prevalence of advanced cases indicate a worse prognosis, lower survival and, consequently, an increase in the mortality rate due to oral cancer (2,3,7,9,11). In this sense, the identification of risk factors denoted in the present study can contribute to the planning of public policies at the susceptible population, aiming to reduce deleterious habits of alcoholism and smoking (3,7,11,23). In addition, knowledge of the factors and most frequent locations of oral cancer will assist dentists, health professionals and the individuals themselves in detecting the lesion at an early stage and, consequently, obtaining a better prognosis (3,11,23).

The present study suggests an association between the higher prevalence of oral cancer in the advanced clinical stage with the anatomical site of the lesion, mainly in the tongue base, and deleterious habits of smoking and alcoholism. This study has limitations with regards the way primary data were collected. In example, the authors did not had access to specific anatomical locations within ICD-10 classifications, as well as there is limited information with regards the amount of alcohol or tobacco each individual used to consumed in the event of diagnosis. Besides that, other relevant information (i.e. HPV infection) is not available within the HRC information system. Although those limitations do not compromise the results or conclusions from this investigation, they puts out the need to collect data that is more specific from diagnosed cases. This would aid a deeper analysis of the prevalence of oral cancer and of its risk factors.

## Conclusions

It is suggested that the localization of malignant neoplasm at the tongue base and the concomitant use of alcohol and tobacco contribute to a higher prevalence of advanced cases of oral cancer. Therefore, it can be suggested that all these characteristics lead to a worse prognosis of oral cancer.
